# Management of Intolerance to Casting the Upper Extremities in Claustrophobic Patients

**DOI:** 10.1155/2014/803047

**Published:** 2014-10-14

**Authors:** Issei Nagura, Takako Kanatani, Masatoshi Sumi, Atsuyuki Inui, Yutaka Mifune, Takeshi Kokubu, Masahiro Kurosaka

**Affiliations:** ^1^Department of Orthopaedic Surgery, Kobe Rosai Hospital, 4-1-23 Kagoike-dori, Chuo-ku, Kobe 651-0053, Japan; ^2^Department of Orthopaedic Surgery, Kobe University Graduate School of Medicine, 7-5-1 Kusunoki-cho, Chuo-ku, Kobe 6500017, Japan

## Abstract

*Introduction*. Some patients showed unusual responses to the immobilization without any objective findings with casts in upper extremities. We hypothesized their that intolerance with excessive anxiety to casts is due to claustrophobia triggered by cast immobilization. The aim of this study is to analyze the relevance of cast immobilization to the feeling of claustrophobia and discover how to handle them. *Methods*. There were nine patients who showed the caustrophobic symptoms with their casts. They were assesed whether they were aware of their claustrophobis themselves. Further we investigated the alternative immobilization to casts. *Results*. Seven out of nine cases that were aware of their claustrophobic tendencies either were given removable splints initially or had the casts converted to removable splints when they exhibited symptoms. The two patients who were unaware of their latent claustrophobic tendencies were identified when they showed similar claustrophobic symptoms to the previous patients soon after short arm cast application. We replaced the casts with removable splints. This resolved the issue in all cases. *Conclusions*. We should be aware of the claustrophobia if patients showed unusual responses to the immobilization without any objective findings with casts in upper extremities, where removal splint is practical alternative to cast to continue the treatment successfully.

## 1. Introduction

Most hand surgeons have experienced seemingly difficult patients who showed excessive anxiety, discomfort, or intolerance to the casting of their upper extremities after surgery without any objective findings pertaining to problems with the casts. Such patients usually repeatedly agitate for cast changes, request the removal of casts insisting on unbearable discomfort and tightness, or cut off the casts by themselves, which can compromise the outcome of the treatment. It is interesting to note that such symptoms rarely occur with lower extremity casts. We hypothesized that some upper extremity patients could have claustrophobia, one of the anxiety disorders characterized by intense fear of enclosed spaces [1]. It can be unpleasant and distressing, but most people who experience the fear find ways to cope, usually through the deliberate avoidance of triggers (like small or enclosed places). Claustrophobic patients do not experience emotional or physiological responses unless they are exposed to a specific stimulus, but they may not always be aware in advance of what stimulus might be a trigger. We recently treated 9 patients in whom claustrophobic symptoms occurred after application of an arm cast following upper extremity surgery. This report details our findings and the relevance of immobilization to their feeling of claustrophobia. Further, we discovered how to identify and deal with such cases.

## 2. Materials and Methods

Between 2009 and 2013, we performed 1574 cases of upper extremity surgeries and we analyzed 9 (5 males and 4 females) of those patients in whom the claustrophobic symptoms of anxiety, discomfort with their casts, and the feeling of excessive tightness combined with an extraordinary squeezing sensation were present after cast application. All patients reported feeling trapped and requested the removal of their casts; however, examination in all cases showed that there was no excessive swelling and progress that was consistent with the nature of their operations. All cases had upper extremity surgery. There were two radius distal fracture cases, two cubital tunnel syndrome cases, two soft tissue tumor cases, one scaphoid fracture case, one extensor pollicis longus injury case, and one carpal tunnel syndrome case. At the time of operation the mean age was 44 (range: 31–56 years of age). In each case, casts had been applied for postoperative immobilization under anesthesia (Table 1). We assessed whether they were aware of claustrophobia themselves and the relevance of immobilization to their feeling of claustrophobia. Further, we investigated how to handle these claustrophobic symptoms.

## 3. Results

Seven out of nine patients were aware of the claustrophobic predisposition and, of these, three immediately requested the use of removable splints rather than casts because they were concerned about their response to a cast. The other 4 patients who were aware of their claustrophobia did not expect a cast would trigger their anxiety but all showed symptoms soon after the cast application. The two patients who were unaware of their claustrophobic predisposition also became distressed soon after cast application. Both cases had previously experienced difficulty when undergoing magnetic resonance imaging (MRI) examination, which reinforced our diagnosis. We suggested to the patients the possibility that their claustrophobic responses might be triggered by the casts and subsequently replaced the casts with removable splints. This resolved the issue promptly.

## 4. Case A

A 44-year-old male was injured with a minimally displaced right clavicle fracture and a left distal radius fracture obtained in a motorcycle accident (Case 1 in Table 1). The left radius fracture was scheduled for surgery. Before the operation, he had not only conceded to have claustrophobia but also requested the application of a removable splint postoperatively because he had recognized the response of his predisposition to a cast. A removable splint was applied after the surgery and his fracture healed well without any trouble. He informed us that the terms “removable” and “skin viewable through the splint” eased his anxiety of being trapped and locked in, which he was then able to rationalize.

## 5. Case B

A 41-year-old woman fractured her right distal radius in a bicycle accident (Case  8, Table 1). A short arm cast was applied after an internal fixation procedure. She reported that the cast was uncomfortable and excessively tight and she was unable to sleep. She kept fidgeting with it and repeatedly removed the bandage under it, which necessitated renewing it at every consultation. Although she was aware that she was unprepared to submit to MRI examination, she did not reconcile this with any claustrophobic tendency. We explained to her our belief that her symptoms might be due to claustrophobia triggered by the cast and subsequently replaced it with a removable splint. Her claustrophobic symptoms ceased and her fracture healed uneventfully. It is likely that the use of a “removable” splint eased her anxiety, because she was able to visualize a part of her hand.

## 6. Discussion

Claustrophobia is one of the most prevalent specific phobias being a fear of enclosed spaces [1]. Tunnels, basement rooms, elevators, subways, and crowded places are all triggers that provoke the fear and people who react to one of these stimuli are likely to react to them all to varying degrees [1]. The incidence of claustrophobia has been reported to be approximately 2 to 13% [2, 3], while the one in our study was 0.5% (1574 of 9 patients).

Lanigan et al. reported 4 cases of claustrophobic symptoms after cast application [4], where neither the authors nor 3 of 4 patients were aware that short arm casts would provoke anxiety. Only one patient admitted being claustrophobic before casting; however, originally all their cases were treated by irremovable casts and multiple cast adjustment was required until being substituted by removable casts. Our experience is similar with a wider range of patients. We found that careful questioning of patients prior to the procedures gave us better outcomes; however, for those patients who were unaware of their claustrophobic tendencies it was difficult to discover their predisposition. If the patient is unwilling to disclose or is unaware of their claustrophobic tendencies even after consultation, an attempt at formal treatment might be required [5]. Generally this involves cognitive therapy that teaches the patient new ways of thinking to control their anxiety [6–8]. Antianxiety drugs have also been used [7]; however, we recommend the application of a removable cast or splint that is applied to give the patient as much visual contact with the immobilized area as possible.

Öst listed “garments that are narrow in the neck” in his claustrophobia scale consisting of 20 questionnaire items because he regarded perceived constriction around neck as a possible trigger for claustrophobic reaction [9]. In his report, some claustrophobics showed increased anxiety or an avoidance reaction to clothing that was tight fit in the neck. It is known that the brain area that receives input from hand abuts the area devoted to the neck [10]. This aligns with our suggestion that any discomfort of tightness, trap, or restriction due to a cast application induces a similar reaction. Further, it has been reported that patients with disability in upper extremity tend to be more affected by psychological factors than patients with disability in some other body parts, which has been reported as psychological reaction of depression, catastrophic thinking to upper extremity disorders [11–14]. Such emotional responses may stimulate a latent claustrophobic predisposition and worsen the symptoms.

In this study, we described claustrophobic patients who reacted to arm casts due to their claustrophobic predisposition. Some patients were not aware of their propensity to this disturbance as previous life experience has not alerted them to the fact. We suggest that it is important for hand surgeons and other associated medical staff to be vigilant in their observation patients particularly in the first few days after immobilization. One should suspect the possibility of claustrophobia if patients show unusual responses and objective findings are absent, particularly with casts of the upper extremities. Then, after consultation and explanation, removable splints should be applied.

We also recommend a screening test for claustrophobia before cast application. Öst [9] and Radomsky et al. [15] developed a claustrophobia questionnaire; however, its application is cumbersome. We are currently developing a simplified questionnaire that will be practical for daily clinical use and will report its performance in the near future.

## Figures and Tables

**Table 1:** 

Case	Sex	Age	Diagnosis	Treatment	Awareness of claustrophobia	Relevance to immobilization
1	M	44	Left radius fracture	ORIF	Yes	Yes
2	M	49	Left cubital tunnel syndrome	Submuscular transposition	Yes	Yes
3	F	31	Left scaphoid fracture	Internal fixation	Yes	Yes
4	M	35	Left elbow soft tissue tumor	Excision	Yes	No
5	F	43	Right hand soft tissue tumor	Excision	Yes	No
6	M	46	Left EPL injury	Tendon repair	Yes	No
7	M	53	Left cubital tunnel syndrome	Submuscular transposition	Yes	No
8	F	41	Right radius fracture	ORIF	No	No
9	F	56	Right carpal tunnel syndrome	Carpal tunnel release	No	No
